# Green Synthesis of Silver Nanoparticles Using Extract of Cilembu Sweet Potatoes (*Ipomoea batatas L var. Rancing*) as Potential Filler for 3D Printed Electroactive and Anti-Infection Scaffolds

**DOI:** 10.3390/molecules26072042

**Published:** 2021-04-02

**Authors:** Arie Wibowo, Gusti U. N. Tajalla, Maradhana A. Marsudi, Glen Cooper, Lia A.T.W. Asri, Fengyuan Liu, Husaini Ardy, Paulo J.D.S. Bartolo

**Affiliations:** 1 Material Science and Engineering Research Group, Faculty of Mechanical and Aerospace Engineering, Institut Teknologi Bandung, Jl. Ganesha 10, Bandung 40132, Indonesia; gusti.unt@lecturer.itk.ac.id (G.U.N.T.); maradhanaa@alumni.itb.ac.id (M.A.M.); lia.asri@material.itb.ac.id (L.A.T.W.A.); husaini@material.itb.ac.id (H.A.); 2 Research Center for Nanoscience and Nanotechnology, Institut Teknologi Bandung, Jl. Ganesha 10, Bandung 40132, Indonesia; 3 Materials and Metallurgy Engineering, Institut Teknologi Kalimantan, Jl. Soekarno Hatta 15, Balikpapan 76127, Indonesia; 4 Department of Mechanical, Aerospace, and Civil Engineering, University of Manchester, Manchester M13 9PL, UK; fengyuan.liu@manchester.ac.uk

**Keywords:** 3D printing, anti-infection scaffold, bone tissue engineering, Cilembu sweet potato, electroactive scaffold, green synthesis, silver nanoparticles

## Abstract

Electroactive biomaterials are fascinating for tissue engineering applications because of their ability to deliver electrical stimulation directly to cells, tissue, and organs. One particularly attractive conductive filler for electroactive biomaterials is silver nanoparticles (AgNPs) because of their high conductivity, antibacterial activity, and ability to promote bone healing. However, production of AgNPs involves a toxic reducing agent which would inhibit biological scaffold performance. This work explores facile and green synthesis of AgNPs using extract of Cilembu sweet potato and studies the effect of baking and precursor concentrations (1, 10 and 100 mM) on AgNPs’ properties. Transmission electron microscope (TEM) results revealed that the smallest particle size of AgNPs (9.95 ± 3.69 nm) with nodular morphology was obtained by utilization of baked extract and ten mM AgNO_3_. Polycaprolactone (PCL)/AgNPs scaffolds exhibited several enhancements compared to PCL scaffolds. Compressive strength was six times greater (3.88 ± 0.42 MPa), more hydrophilic (contact angle of 76.8 ± 1.7°), conductive (2.3 ± 0.5 × 10^−3^ S/cm) and exhibited anti-bacterial properties against *Staphylococcus aureus* ATCC3658 (99.5% reduction of surviving bacteria). Despite the promising results, further investigation on biological assessment is required to obtain comprehensive study of this scaffold. This green synthesis approach together with the use of 3D printing opens a new route to manufacture AgNPs-based electroactive with improved anti-bacterial properties without utilization of any toxic organic solvents.

## 1. Introduction

Inspired by the nature of the human body and the tiny electrical current present within them, electrical stimuli has emerged as one of the most promising types of external stimulation methods that can be used to enhance the rate of tissue regeneration and modulate cellular specialization [1,2,3]. The success of electrical stimulation therapy alongside tissue engineering is accompanied by the development of electroactive biomaterials, which is designed to be used as tissue engineering scaffolds with the ability to directly deliver electrical, electrochemical, or electromechanical stimulations directly into the cells, tissues, or organs [1,2]. Electroactive scaffolds have been successfully used for bone [4,5,6], neural [1,7], muscles [8], and cardiac tissue engineering applications [9,10]. In particular, low voltage direct current electrical stimulation has been used clinically in bone healing treatments as a complement to standard fracture care, and has been shown to greatly support the osteogenesis process [4].

Polymeric-based materials have been shown to be a prime candidate for bone tissue engineering applications, due to their highly flexible and tailorable properties, ease of fabrication, excellent biocompatibility, and the possibility to be degraded over time in physiological media [11]. One strategy to fabricate electroactive scaffolds is by using an intrinsically conductive polymer as its sole material, although currently available options are fairly limited as they require the presence of conjugated π-electron backbone. Thus far, the most widely used conductive polymers are poly [3,4-(ethylenedioxy)thiophene] (PEDOT) [7], polypyrrole (PPy) [5] and polyaniline (PANI) [6,12]. However, the poor processability, non-biodegradability, unsatisfactory mechanical properties, and potential cytotoxicity of these polymers have limited their use as a sole component in biomedical applications [11]. Another strategy is to create a composite by embedding electrically conductive particles, the filler, into a polymeric matrix. This allows the formation of a continuous network of conductive filler throughout the polymer once the volume fraction of the filler in the matrix has reached a certain percolation threshold [11]. Formation of such filler networks essentially means that the filler will handle all electrical conducting activity, allowing the usage of more processable, mechanically superior, biocompatible, and biodegradable non-conductive polymers such as polycaprolactone (PCL) [6,9] and poly(lactic acid) (PLA) [12,13,14,15]. Commonly used conductive filler materials include carbon-based materials [3,8,16,17], conductive polymers [6], and metallic-based materials [10]. It has been reported that reduction in the percolation threshold can be achieved by using nanoparticles instead of microfillers [16]. With the recent advancements in the field of nanotechnologies, metallic materials can now be synthesized in their nanoscale form, thus making metallic-based materials more attractive as a conductive filler.

Among many contenders, silver nanoparticles (AgNPs) are an attractive material for metallic-based conductive filler due to their high conductivity (2.2 × 10^4^ S/cm) [18], ability to promote bone healing by stimulating cell proliferation, and osteogenic differentiation [19,20,21], as well as their ability to disrupt the formation of microbial biofilm [22] and potentially overcome multidrug-resistant bacteria [23]. These unique properties made AgNPs particularly desirable for use in biosensors [24], prostheses [25], wound healing [26], and dental materials [27]. However, the conventional synthesis method of AgNPs often involves the usage of toxic chemical reducing agents which are hazardous for humans (e.g., sodium borohydride, hydrazine), causing the resulting AgNPs to be deemed unsafe for biomedical use [28,29]. In an attempt to solve these issues, “green synthesis” methods have emerged as a viable and promising option to fabricate AgNPs, offering simple, cheap, rapid, and non-toxic premises [19,28,30,31,32]. Instead of using chemical reducing agents, green synthesis methods utilize various biological entities such as plant extract [33,34,35], bacteria [36], and fungi [37] as both reducing and capping agents to produce a high yield of relatively uniform-sized metallic nanoparticles. In particular, plant-based extract has been receiving increased attention due to its accessibility, safety, as well as ease of synthesis, as they do not require any maintenance or preservation of microbial culture [38]. Thus far, utilization of leaf and fruit extracts of various plants are the most commonly reported. Among many plants, sweet potato is an interesting candidate for this purpose, not only because it contains high concentration of various reducing agents (e.g., reducing sugars, phenolic compounds, vitamin C, β-carotene), but also because it has a lot of unexplored regional variants, each offering slightly different compositions to one another [39,40,41,42].

Herein, we report a simple green synthesis approach towards AgNPs production using ‘Cilembu’ sweet potato (*Ipomoea batatas L var. Rancing*) extract. Native to the West Java region, Indonesia, this particular cultivar of sweet potato offers one of the highest pre-baked starch contents out of all variants of sweet potatoes. When baked at temperature of 180–200 °C for 60 to 90 min, the starch will degrade into maltose—a reducing sugar—contributing to its honey-like sweetness [43]. The high maltose content of baked ‘Cilembu’ sweet potato (14.88 ± 0.44%) [44] relative to the average of other sweet potato cultivars (8.81~13.97%) [43] has piqued our interest on the possibility of using said extract as an effective reducing and capping agent for AgNPs green synthesis. This work will address the effect of varying the Ag precursor concentration and baking process of the sweet potatoes before extracting to convert the starch into maltose. Both of these parameters will significantly affect the resulting AgNPs size, distribution, crystalline phase identity and antibacterial activity. Its potential as an electroconductive and antibacterial filler in polymeric bone scaffolds will also be assessed. Based on our previous research, PCL was chosen as the polymer matrix material, and 3D printing was chosen as the fabrication method [6]. This study aims to evaluate the physical, chemical, electrical, and antibacterial properties of a 3D-printed electroactive and anti-infection PCL/AgNPs composite scaffold manufactured using green synthesis.

## 2. Results and Discussion

### 2.1. Green Synthesis of AgNPs

#### 2.1.1. Visual Observation and UV-Vis Spectroscopy Results

**Figure 1:**
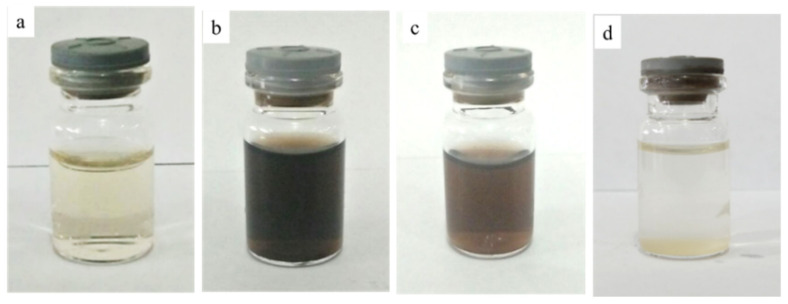
Visual appearances of AgNPs colloidal samples: (**a**) Baked 1, (**b**) Baked 10, (**c**) Baked 100 and (**d**) Unbaked 10.

The formation of AgNPs is indicated by a color change from colorless AgNO_3_ solution to dark brown. Figure 1 shows the visual appearance of all synthesized colloidal samples. The presence of black colloidal particles was observed when baked extract was combined with ten mM and 100 mM of AgNO_3_ (namely Baked 10 and Baked 100 respectively). The sample’s solution with one mM of AgNO_3_ (denoted as Baked 1) remains colorless due to low Ag precursor content, producing only a minuscule amount of AgNPs. Samples made using unbaked extract (namely Unbaked 10) also remain transparent, possibly due to the lack of reducing sugar responsible for reducing Ag^+^ to Ag^0^. This phenomenon can occur because the baking process promotes maltose formation, which can significantly increase maltose content from negligible to >50% (of the total sugar composition of the sweet potato) [43]. Some of the precipitates were seen on the bottom of the vial in the Baked 100 sample, suggesting that some of the black colloidal particle’s agglomeration might occur in this sample. Agglomeration might explain why the color of the Baked 100 solution is lighter than the Baked 10 solution.

**Figure 2:**
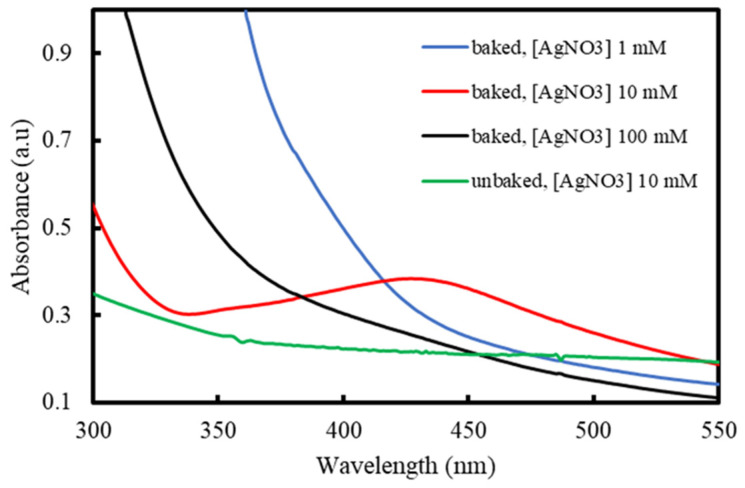
UV-Vis spectra of the colloidal samples.

Spherical AgNPs exhibit UV-Vis peak in the 400–500 nm range due to their surface plasmon resonance (SPR) characteristic [45]. The Baked 10 sample demonstrated a broad peak centered at 428 nm (Figure 2), further supporting the visual data regarding the presence of AgNPs in the colloidal solution. Similar peaks were not observed in the other samples, indicating that AgNPs are absent. From their visual appearances, the Unbaked 10 and the Baked 1 samples were not expected to contain any AgNPs. The lack of any relevant peak in the Baked 100 sample can be attributed to the presence of silver metal above the nanosized scale despite the black visual appearance of the sample. As previously reported by Ahmadi et al., higher AgNO_3_ concentrations increase the chance of Ag collisions due to the inadequate amount of capping agent amount relative to the produced AgNPs’ total volume [34]. These collisions lead individual AgNPs to become agglomerated, significantly increasing their particle size, causing them to become undetected when exploiting their SPR property using UV-Vis.

#### 2.1.2. X-ray Diffraction (XRD) Results

**Figure 3:**
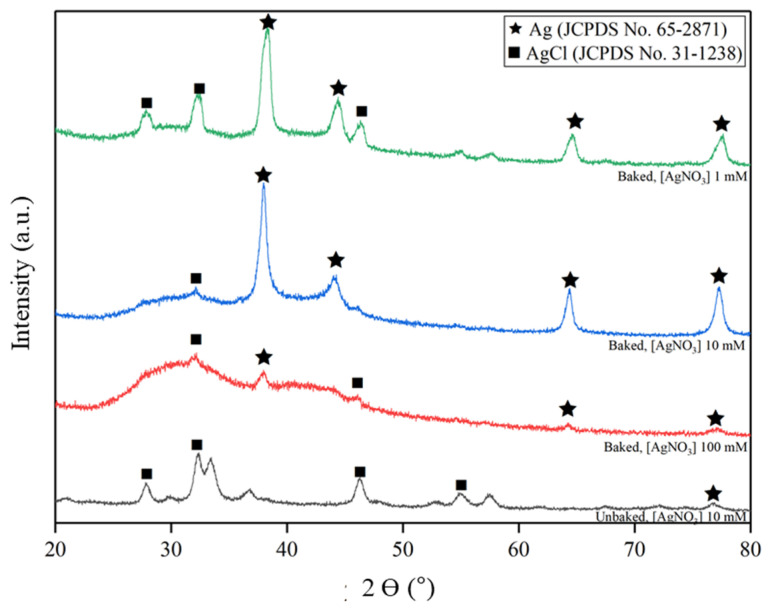
XRD patterns of samples powder prepared at various extract type and AgNO_3_ concentration.

The crystalline phase of the nanoparticles was studied by XRD, and the result is shown in Figure 3. All the baked samples showed dominant peaks at 38°, 44°, 64° and 77° that belong to silver metal with FCC lattice (Ag; JCPDS No. 65-2871) of orientation (111), (200), (220), and (311) respectively. In contrast, peaks at 28°, 32°, 46°, and 55° were observed in the unbaked sample, which are attributed to chlorargyrite (silver chloride; AgCl; JCPDS No. 31-1238) of orientation (111), (200), (220), and (311) respectively. From these results, it can be inferred that baking process, which dramatically increases the maltose content as reducing sugar, is crucial in assisting the formation of AgNPs. Broad peaks found in higher AgNO_3_ concentrations, particularly in the Baked 100 sample, indicates a more amorphous structure, possibly due to the rapid reaction kinetics that related to higher reactant concentration.

To understand why AgCl was formed in differing quantities, ion chromatography was carried out to determine Cl^−^ ion concentration found in both baked and unbaked sweet potato extract. It was found that the average concentration of Cl^−^ ion in both extracts is 2.91 ± 1.55 × 10^−3^ M, with negligible variation by the introduction of the baking process. Referring to our visual, UV-Vis and XRD data, it can be assumed that minimal reduction process happened in the Unbaked 10 sample. Since the equilibrium constant (Q) of the samples with AgNO_3_ concentrations 10 mM (Q = 2.91 ± 1.55 × 10^−5^) are greater than the solubility constant of AgCl (Ksp = 1.77 × 10^−10^) [46], the formation of AgCl is favorable when a higher quantity of free Ag^+^ ions is present in the solution. Therefore, it is reasonable that AgCl was formed in the Unbaked 10 sample due to the absence of reducing agent (maltose) in the unbaked extract. The existence of AgCl in the Baked 100 sample was also detected, possibly due to the lack of a reducing agent against a high concentration of Ag^+^ in solution, resulting in a high concentration of free non-stabilized Ag^+^. The lowest amount of AgCl impurities were found in the Baked 10 sample, as it contains the highest amount of stabilized AgNPs according to UV-Vis spectra.

A unique case can be seen in the Baked 1 sample, as it has a higher intensity of AgCl than the Baked 10 sample. It is likely that due to the excessive amount of extract available relative to the silver precursor, some starch managed to encapsulate unreduced Ag^+^ ions. However, as starch does not present any aldehyde functional groups able to reduce Ag^+^ ions, they remained as positively charged ions, forming starch- Ag^+^ complexes [47]. As the Cl^−^ ions present a tiny ionic radius (1.81 Å) [48], we can assume that the Cl^−^ ions managed to infiltrate the gaps between starch molecule chains and bond with the free unreduced Ag^+^ forming AgCl. A similar case does not happen in the Baked 10 sample, as the ratio between extract and silver precursor was not excessive.

#### 2.1.3. Dynamic Light Scattering (DLS) and Zeta Potential Results

**Figure 4:**
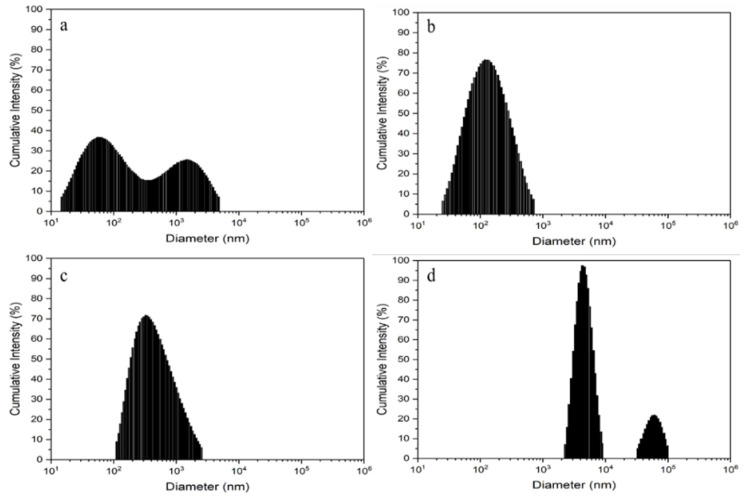
Intensity distribution of particles in the colloidal samples: (**a**) Baked 1, (**b**) Baked 10, (**c**) Baked 100 and (**d**) Unbaked 10.

Figure 4 shows the histogram particle size distribution of each samples in a logarithmic scale and the DLS results are summarized in Table 1. As observed, the Baked 10 sample exhibits the lowest initial average particle size, followed by Baked 1, Baked 100, and Unbaked 10 in increasing order of average particle size. The sample containing unbaked extract has by far the largest average particle size, which can be attributed to the formation and agglomeration of AgCl due to the lack of appropriate biological moiety (i.e., maltose) available to reduce and stabilize Ag^+^.

To predict the stability of colloidal particles and potential agglomeration that might occur in colloidal samples, zeta potential measurement was performed. As can be seen in Table 1, the Baked 10 sample has the highest zeta potential (−41.0 ± 9.0 mV), while the Unbaked 10 sample has the lowest zeta potential (−0.3 ± 0.1 mV). In general, higher absolute value of zeta potential will lead to higher stability of the colloidal particles [30,49]. AgNPs in the Baked 10 sample is considered stable and able to maintain their small particle size (105.5 ± 12.6 nm) because its zeta potential (−41.0 ± 9.0 mV) is more negative than −30 mV. Early suspension of AgNPs was indicated in the Baked 1 sample because its zeta potential (−15.5 ± 2.0 mV) is at the agglomeration threshold (±15 mV). Rapid coagulation or flocculation of colloidal particles leading to the formation of big particles might have occurred in the Baked 100 (456.5 ± 14.7 nm) and the Unbaked 10 (5958.8 ± 499.6 nm) samples, because their zeta potentials (−0.7 ± 0.4 mV for the Baked 100 and −0.3 ± 0.1 mV for the Unbaked 10 sample respectively) are in the range of 0 ± 3 mV [49].

#### 2.1.4. Transmission Electron Microscopy (TEM) Results

**Figure 5:**
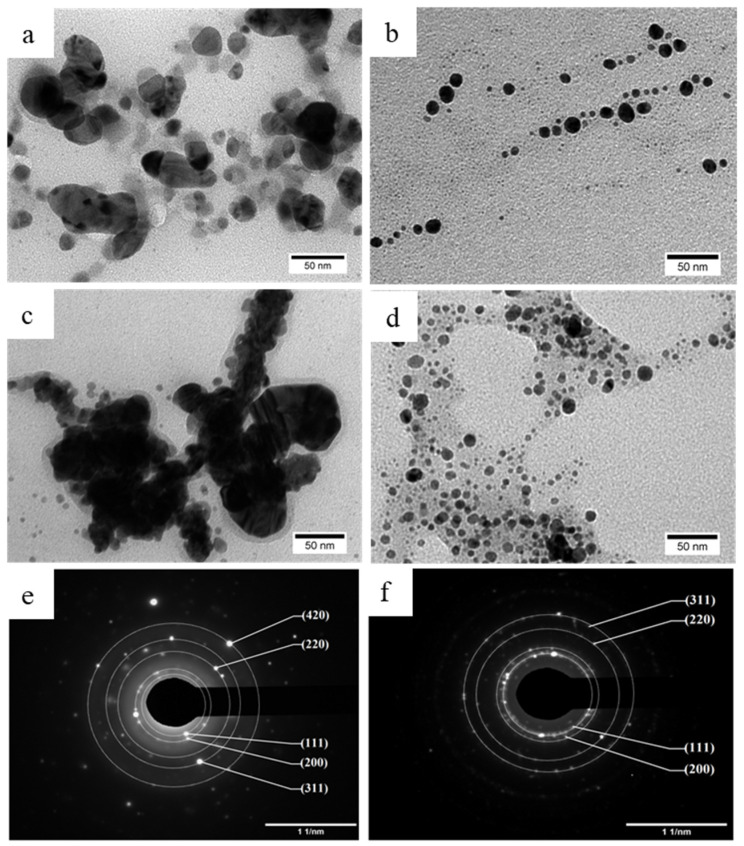
TEM images of the resulting AgNPs from colloidal samples: (**a**) Baked 1, (**b**) Baked 10, (**c**) Baked 100 and (**d**) Unbaked 10. Selected area electron diffraction (SAED) results of: (**e**) Baked 10 and (**f**) Unbaked 10 samples.

TEM analysis was carried out to understand the causes of the size difference between the three baked samples with differing AgNO_3_ concentrations and the results are shown in Figure 5. Nanoparticles detected in the Baked 1 sample have irregular morphology and size (26.44 ± 11.98 nm) and are encapsulated with translucent coating layer, with some entities connected to each other (Figure 5a). Nanoparticles that were observed in the Baked 10 sample (Figure 5c) are uniform with nodular morphology (9.95 ± 3.69 nm), which is slightly smaller on average than previous report utilizing similar AgNO_3_ concentration with different cultivar of potato [50]. Increasing the concentration of AgNO_3_ to 100 mM (the Baked 100 sample; Figure 5c) results in agglomerated particles with a thin coating layer encapsulating them as a single entity (326.7 ± 19.1 nm). The ratio between extract and AgNO_3_ concentration plays an integral role in defining the average particle size of AgNPs. Low levels of baked extract relative to the concentration of Ag^+^ ions means that the reduction reaction will happen slowly, as there will only be a small number of nucleating sites. Slow reaction time promotes particle growth, resulting in a small number of large-sized individual nanoparticles [51]. As the ratio increases, the reaction time becomes faster, and more nucleation can coincide, resulting in a more significant number of smaller non-agglomerated individual nanoparticles.

It is noteworthy that the hydrodynamic diameter of coated nanoparticles that was measured by DLS is the combined result of the core inorganic particles and the hydrated outer capping layer [52]. In contrast, the real particle size of coated nanoparticles that was determined by TEM images is only correlated with the core inorganic particles that are highly visible in TEM. Therefore, it is reasonable that the average particle size results from the DLS results are bigger than the TEM images of the coated nanoparticles in the colloidal samples. This tendency was also observed by Hileuskaya et al. in pectin-capped AgNPs, where particle size from DLS results (in the range of 63–435 nm) are more than ten times bigger than particle size from TEM images (in the range 8–28 nm) [22].

TEM observation also revealed that the use of unbaked sweet potato extract results in a very thick coating layer which encapsulates and connects many nanoparticles as one single giant entity (Figure 5d). Considering that polymers and organic content have low contrast in TEM observation due to their low-mass composing elements [53], the coating layer is suspected to be starch, forming a starch-Ag complex, as starch is the main form of sugar present in the unbaked sweet potato [43]. The coating layer in the Unbaked 10 sample is significantly thicker than the Baked 10 sample because starch content in unbaked is larger than baked sweet potato extracts [43]. To know the crystalline phase of black particles that were observed in TEM images of baked and unbaked samples, further characterization using selected area electron diffraction (SAED) was performed on the Baked 10 sample, as representative of the Baked samples, and the Unbaked 10 sample. SAED results unveiled that the black particles in the Baked 10 sample (Figure 5e) are associated with Ag (JCPDS No. 65-2871), while the black particles in the Unbaked 10 sample (Figure 5f) belonged to AgCl (JCPDS No. 31-1238). These results are consistent with XRD results and confirm the crucial role of the baking process in elevating maltose content on the formation of AgNPs.

Morphology and particle size of AgNPs may affect their antibacterial activity. Spherical-shaped AgNPs display better antibacterial activity than triangular, linear, and cubic AgNPs due to their larger surface-to-volume ratio [19,54]. AgNPs also exhibit higher antibacterial activity by decreasing their particles size [19,23]. Based on TEM observation about morphology and particle size of obtained AgNPs, it could be expected that the antibacterial activity of AgNPs in the Baked 10 sample might be better than other samples due to its spherical-shaped morphology and lowest particle size (9.95 ± 3.69 nm).

#### 2.1.5. Fourier Transform Infrared (FTIR) Results

**Figure 6:**
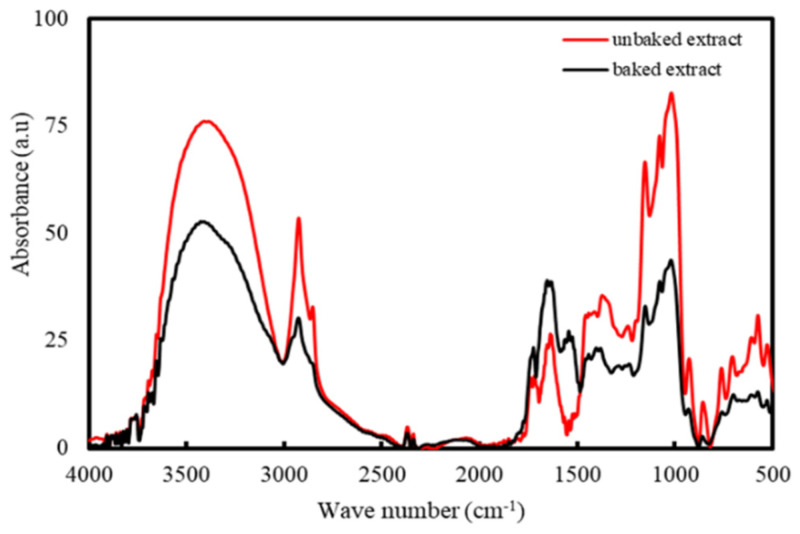
FTIR spectra of the unbaked and baked extract of Cilembu sweet potatoes.

Figure 6 shows the results from FTIR. The dominant absorbance peaks are seen at 3427 and 1633 cm^−1^ present in both samples may be attributed to the O-H stretching of aromatic compounds and N-H stretching in primary and secondary amines, as well as C=O stretching of amides, respectively [50]. The C-H bending of alkanes and alkenes corresponds to the peaks at 1373 and 1022 cm^−1^, respectively [55]. The peak at 1726 cm^−1^ corresponds to the stretching vibration of the C-N bond present in amine I [35]. The peak at 1153 cm^−1^ corresponds to C-O found in aldehyde [55]. The emergence of a new small peak in the 1544 cm^−1^ after baking may be attributed to the bending of the C-H bonds present in hydrocarbons [50].

**Figure 7:**
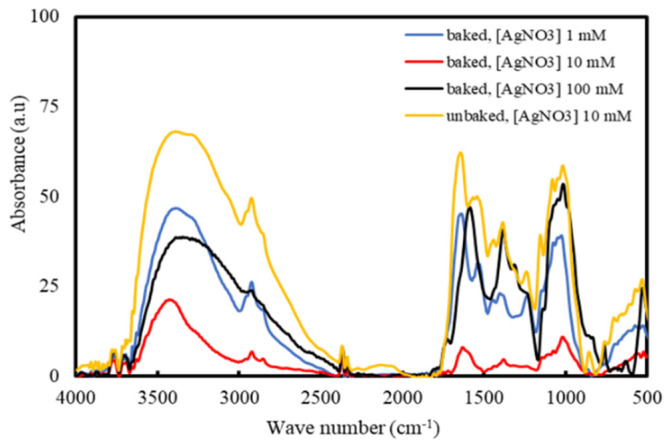
FTIR spectra of the colloidal samples.

The comparison between the FTIR spectra of pristine extract (Figure 6) and the produced AgNPs (Figure 7) allow for the determination of the compounds that are responsible for the reduction of Ag ions and the stabilization of AgNPs. As observed from Figure 6 and Figure 7, the peaks corresponding to the C-N in amine I (1726 cm^−1^) and the C-O in aldehyde (1153 cm^−1^) experiences broaden and reduce in intensity. A new peak emerges at 1543 cm^−1^, which may belong to a nitrate ion due to AgNO_3_ reduction [56]. The shifting of these peaks could be attributed to the interaction of related functional groups with the AgNPs [36]. Amide linkage present in protein has a high probability of linking with silver, which will then cause the protein to deposit around the silver, encapsulating the AgNPs. Therefore, it is likely that the synthesized AgNPs are capped and stabilized by compounds containing amine and aldehyde groups. Aromatic compounds such as ascorbic acid, riboflavin, other phenolic compounds, as well as proteins that are naturally present in Cilembu sweet potato are suspected to be the compounds responsible for the stabilization of the silver nanoparticle in the aqueous medium [57].

#### 2.1.6. Antibacterial Properties

**Figure 8:**
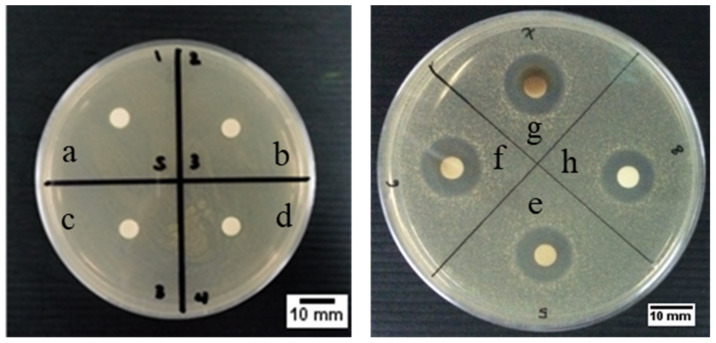
The representative of antibacterial testing result using the disc diffusion method of (**a**) extract of baked Cilembu sweet potatoes, powder of: (**b**) Baked 1, (**c**) Baked 10, (**d**) Baked 100. Colloidal sample of: (**e**) Baked 1, (**f**) Baked 10, (**g**) Baked 100 and (**h**) Unbaked 10.

The disc diffusion testing method was used to qualitatively confirm the antibacterial properties of our samples against common infection-causing model bacteria (*Staphylococcus aureus* ATCC 3658). The visual and statistical data of the disc diffusion results of our samples are reported in Figure 8 and Table 2, respectively. This gram-positive bacteria was chosen as infection-causing model bacteria in this study because Staphylococci (*S. aureus* and *S. epidermidis*) are the primary causative agent of infection in orthopedics, with roughly 80% of implant-related infection caused by this bacteria [58,59]. Furthermore, Lyutakov et al. found that antibacterial activity of polymethylmethacrylate (PMMA) thin film doped AgNPs seems to be more effective against gram-negative bacteria (*Escherichia coli*) than gram-positive bacteria (*S. epidermidis*) [60]. Therefore, utilization of *S. aureus* will likely be a sufficient test to determine the antibacterial performance of our AgNPs samples.

As can be seen in Figure 8a, the absence of zone of inhibition (ZOI) was observed in the extract-only sample, indicating that the extract alone does not possess any antibacterial activity on its own. No clear ZOI were also observed in the powder form of AgNPs samples (Figure 8b–d), indicating the lack of sufficient bactericidal activity of these samples. Generally, bulk particles in powder samples will elute Ag^+^ in a much slower rate than nanoparticles in colloidal samples due to the lower solubility. Thus, powder samples are unsuitable for disc diffusion test to provide significant antibacterial information in such a short duration test (24 h) due to their slow Ag^+^ release. To comply with such limitations, colloidal samples were used instead, and the results were much clearer and quantifiable (Figure 8e–h).

The measured colloidal samples’ ZOI is shown in Table 2. It is evident from the three baked samples that increasing the Ag precursor’s concentration will lead to higher antibacterial activity, as more Ag^+^ ions will be available to cause cellular destruction. Baked sample (the Baked 10; ZOI 12.9 ± 0.3 mm; Table 2) displays noticeably higher antibacterial activity than its unbaked counterpart with similar AgNO_3_ concentration (the Unbaked 10; ZOI 11.7 ± 0.3 mm; Table 2). One possible explanation for heightened activity in the Baked 10 sample can be attributed to its smaller and more dispersed individual particles. In contrast, the Unbaked 10 sample consists of many individual particles joined together by a thick encapsulating layer (TEM images, Figure 5b and Figure 5d respectively). Smaller particle size were known to enhance antibacterial performance, as there will be a more significant number of atoms on the surface that can readily interact with the bacteria and release silver ions to kill the bacterial cells [19,23]. Since all of our colloidal samples have antibacterial properties and antibacterial properties of the Baked 10 sample are higher than the Unbaked 10 sample at the same precursor (AgNO_3_) concentration, AgNPs from the Baked 10 sample was used for preparing a 3D printed PCL/AgNPs scaffold.

### 2.2. Fabrication of 3D Printed Scaffold

#### 2.2.1. Scaffold Morphology and Porosity

**Figure 9:**
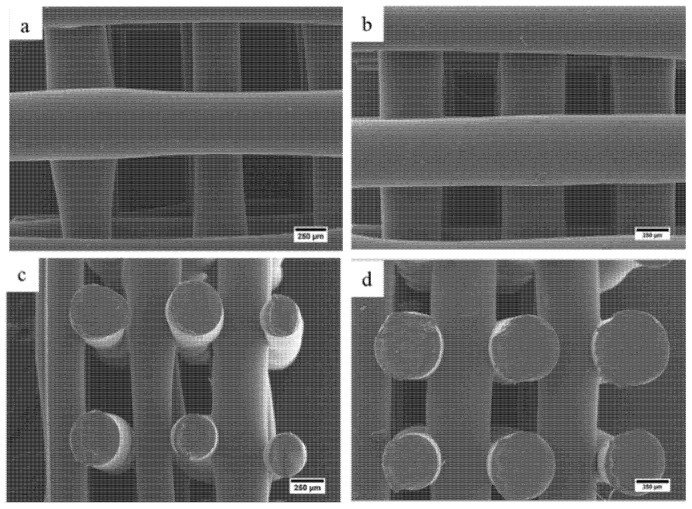
SEM images of fabricated scaffold from top-view: (**a**) PCL and (**b**) PCL/AgNPs. Side-view: (**c**) PCL and (**d**) PCL/AgNPs.

SEM was used to analyze the scaffold morphology and to quantify the pore size and the fiber diameter (Figure 9 and Table 3). As observed, scaffolds present uniform distributed pores with regular shapes without significant differences in pore size (~500 μm) and fiber diameter (~500 μm) compared to the initially designed values. Results also show that the addition of AgNPs slightly increase the filament diameter, decreasing pore size. The scaffolds’ porosity ranges between 60 and 70% (higher values in the case of PCL scaffolds) making them suitable for bone tissue engineering applications.

#### 2.2.2. Wettability

**Figure 10:**
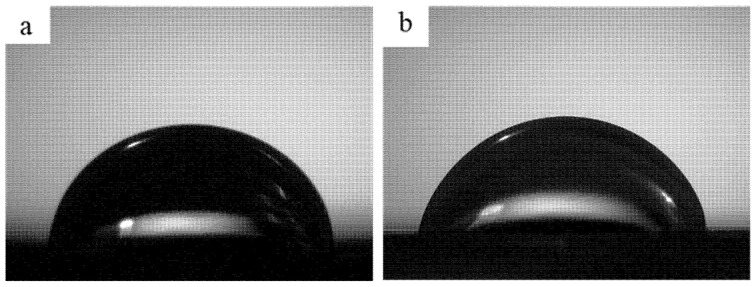
The representative of contact angle images (3 s) of the water droplet on: (**a**) PCL scaffold, (**b**) PCL/AgNPs scaffold.

Another important parameter to ensure scaffold’s biocompatibility is wettability because it affects the interaction between biological molecules or cells and biomaterial surface. Thus, the scaffolds’ wettability was determined by water contact angle measurement (Figure 10).

As observed, the contact angle of the 3D printed PCL scaffold is 80.6 ± 2.0°, which is similar to the previous report by Liu et al. using similar scaffold design (80.9 ± 2.7° [61]). The addition of 0.5 wt.% of AgNPs on the 3D printed PCL scaffold induces a slightly decrease in the scaffold’s contact angle (76.8 ± 1.7°). This result is consistent with the previous report by Liu et al. that the addition of 2–25 wt.% of AgNPs on PLA/18% nano hydroxy apatite (nHA) composites reduces the contact angle from 80.9 ± 2.2° to 77.4 ± 2.5° and 33.6 ± 5.3°, respectively [13]. Considering that scaffold with a lower contact angle is favorable for cell attachment due to its hydrophilic characteristic, the addition of 0.5 wt.% of AgNPs on PCL scaffold leads to better cell attachment on the scaffold.

#### 2.2.3. Mechanical Properties

**Figure 11:**
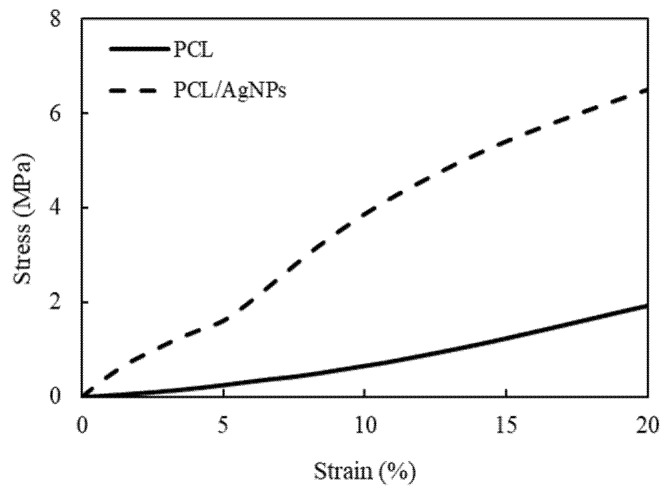
Representative compressive stress–strain curves of the 3D-printed PCL and PCL/AgNPs scaffolds.

Even though polymeric-based scaffolds are considered one of the best candidates for bone tissue engineering, its application is still hindered by its relatively low mechanical properties. One way to mitigate this shortcoming is by incorporating filler particles into the polymer matrix, essentially creating a composite. In this study, mechanical properties of the scaffold were evaluated by uniaxial compression (Figure 11), and the compressive Young’s modulus and strength were determined based on ISO 844-2014 standard for testing of rigid cellular plastics [62] (Table 4).

As shown in Table 4, the addition of 0.5 wt.% AgNPs to the PCL scaffold significantly enhanced their compressive mechanical properties. Compressive strength of PCL/AgNPs scaffold (3.88 ± 0.42 MPa) is about six times higher than PCL scaffold (0.65 ± 0.09 MPa), while compressive Young’s modulus of PCL/AgNPs scaffold (52.42 ± 7.64 MPa) is 3.8 times higher than PCL scaffold (13.65 ± 2.17 MPa). The introduction of Ag nanoparticles to PCL matrix can be seen as a means to fabricate particle-reinforced composite. In a particle-reinforced composite system, the particles (AgNPs) will act as the load-carrying medium, while the matrix (PCL) will act as the load transporting medium. The previous report concluded that a strong interaction force between PCL and AgNPs interface contributes to heightened stiffness/Young’s modulus [63]. Increment of mechanical properties of polymeric scaffold after addition of filler was observed not only in PCL/AgNPs composite, but also in other materials with similar concepts such as PCL/multi-walled carbon nanotubes (MWCNT) [17], PCL/PANI [6], and Chitosan/Ag [64] bone scaffolds. In addition, the incorporation of AgNPs was also shown to reduce the scaffold’s porosity from 70 ± 34% (PCL scaffold; Table 3) to 60 ± 4% (PCL/AgNPs scaffold; Table 3), which is known to be inversely proportional to the scaffold’s compressive strength (i.e., scaffold compressive strength will increase when scaffold porosity decreases) [65].

It is well known that the mechanical properties of human cancellous bone can be quite diverse, with compressive strength ranging from 1–12 MPa and Young’s modulus ranging from 100–5000 MPa [66]. According to this report, mechanical properties of the fabricated PCL/AgNPs scaffold may fit in the lower range of human cancellous bone, making it suitable for use as scaffold for cancellous bone applications.

#### 2.2.4. Conductivity

As PCL is a non-conductive polymer, it is necessary to add a conductive filler (AgNPs in this study) for the fabrication of electroactive scaffolds. The conductivity of the scaffolds was measured using the four-point probe method. PCL’s conductivity has been shown to be approximately 1.1 × 10^−11^ S/cm at 20 ± 2 °C [67]. Results showed that the inclusion of 0.5% wt. of AgNPs significantly increased the scaffold conductivity (2.25 ± 0.54 × 10^−3^ S/cm). The conductivity of PCL/AgNPs scaffolds is higher than conductivity of cancellous (1.6–2.0 × 10^−3^ S/cm) and cortical bone (5.8–6.3 × 10^−4^ S/cm) [68]. The addition of AgNPs improves the printed scaffold’s conductivity and is potentially suitable for applying electrical stimulation to guide cell behavior, as the scaffold has higher conductivity stimulation could be achieved with lower voltage.

#### 2.2.5. Antibacterial Properties

**Figure 12:**
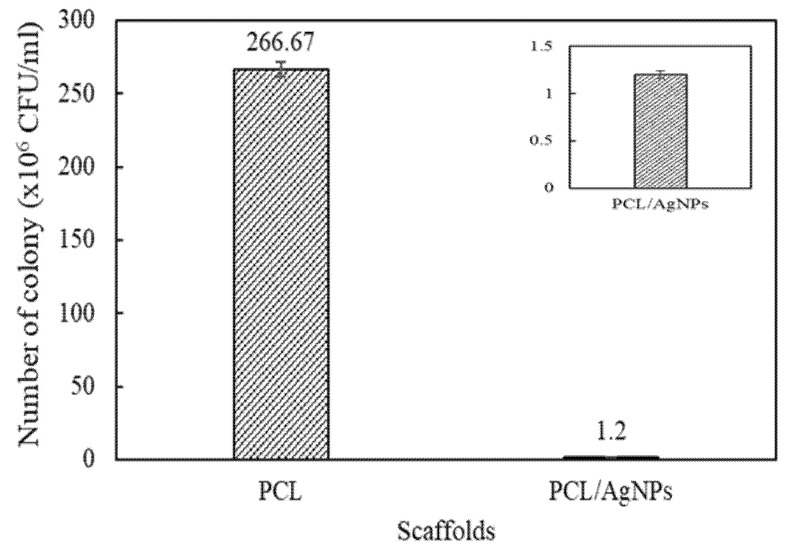
Antibacterial testing result of scaffolds using total plate count (TPC) method.

The presence of AgNPs in the scaffold is expected to provide anti-infection capability against infection-causing bacteria during its period of in vivo implantation. Thus, it is important to evaluate the fabricated scaffold’s antibacterial properties against common infection-causing model bacteria (*S. aureus*; ATCC 3658). Considering that solid samples could not provide significant ZOI information at a short duration, results from a test of the disc diffusion method were presented in Figure 8b–d, while the scaffold’s antibacterial properties were quantitatively described by the total plate count method (Figure 12).

As can be seen in Figure 12, the number of surviving bacterial colonies in the PCL scaffolds and the PCL/AgNPs scaffolds are 266.7 ± 5.1 × 10^6^ CFU/mL and 1.2 ± 0.04 × 10^6^ CFU/mL respectively. Thus, the addition of 0.5 wt.% AgNPs to the scaffolds significantly reduces the number of surviving bacterial colonies to only 0.5% of surviving bacteria after 24 h incubation, highlighting the PCL/AgNPs scaffold’s excellent antibacterial activity.

### 2.3. AgNPs Production and Scaffold Fabrication Discussion

The development of a green synthesis method using biological entities, including plant extract as reducing and capping agent, has opened up the possibility of generating minimally toxic AgNPs via a simple, cheap and environmentally friendly method. This work has reported the creation of AgNPs by green synthesis approach using the extract of Cilembu sweet potato as a green mediator. The obtained AgNPs were successfully utilized as filler materials in PCL/AgNPs electroactive and anti-infection bone scaffold that was fabricated by 3D printing method.

Previous demonstrations of green synthesized AgNPs using potato extract by Roy et al. resulted in spherical AgNPs with 10–12 nm diameter [50], which is further confirmed by Hileuskaya et al., reporting generation of spherical AgNPs with 8–28 nm by using pectin [22]. These results are in line with our findings, particularly AgNPs from the Baked 10 sample as our optimal sample, which has spherical shape with comparable particles size of ~9.95 nm. The comparatively small size of the synthesized AgNPs may be attributed to the high maltose content as reducing agent and the necessary capping agents’ presence in the extract of baked Cilembu sweet potato. Previous reports showed that AgNPs with spherical shape [19,54] and small particle size [22,36], especially when the size is less than 10 nm [19], have superior antibacterial activity. Thus, it could be predicted that AgNPs from the Baked 10 sample have excellent antibacterial properties, which is implied from the high antibacterial activity against *S. aureus* as infection-causing model bacteria. However, more studies are needed to assess its antibacterial activity against other strains of bacteria.

Even though our AgNPs show high antibacterial activity against *S. aureus*, their antimicrobial mechanism remains unclear. There are several antibacterial actions of AgNPs that have been reported in the literature, the first of which are that AgNPs could attach and penetrate the bacterial cell membrane, leading to direct damage of the cell membrane [19,22,36,49]. Also, it is well known that silver nanoparticles can undergo oxidative dissolution when exposed to H_2_O_2_ or O_2_,—which can be found inside bacterial cell membranes—generating silver ions in vivo. It is hypothesized that the generated Ag^+^ can interact with several different components in bacterial cells to induce cellular destruction (i.e., by binding with thiol group-containing respiratory enzyme, transport protein, and DNA, among others) [19,49]. Considering that deeper insight about antibacterial mechanism of our AgNPs is important for their application, further studies are essential to evaluate antibacterial mechanisms of our AgNPs.

The AgNPs were used successfully as filler material in 3D printed tissue scaffolds with fiber diameter and pore size of 536 μm and 425 µm, respectively. This was a reduction in pore size of around 25% compared to pure PCL scaffolds. These morphological changes are similar to those observed by other groups who manufactured PCL/AgNPs scaffolds [69]. Although these changes are not expected to greatly affect the biological properties of the scaffold, manufacturing parameters such as melt temperature, extrusion pressure, and nozzle speed could be adjusted to alter the morphology of the PCL/AgNPs scaffolds to the most desired geometries. AgNPs addition to the scaffold is likely to increase the biocompatibility, as indicated by the slight decrease in contact angle from the wettability test, similar to observations in previous studies [61]. Additionally, mechanical strength was increased by six times compared to the pure PCL scaffold but some of this will be due to the reduction in porosity due to the increase in fiber diameter for the PCL/AgNPs scaffolds. However, increases in mechanical strength have been observed by other groups who used AgNPs fillers [63].

The fabricated 3D PCL/AgNPs scaffold showed very promising antibacterial properties which could be present both on implantation and over a longer period whilst the scaffold degrades. This finding is in line with Gao et al., where the addition of 1 wt.% AgNPs managed to reduce bacteria survival to 32.2% over a four h incubation period [70]. When incorporated in a scaffold, AgNPs may come into direct contact with the bacterial cellular membrane, generating Ag^+^ ions in the process to annihilate the bacteria without the need for the AgNPs to penetrate the bacterial cell [19]. It is worth noting that antibacterial activity of AgNPs is lower than Ag^+^, as reported by Lyutakov et al. [60]. However, AgNPs may provide long term antibacterial activity because it takes two months in static conditions to completely release all AgNPs from PMMA thin film, while the efficacy of Ag^+^ is only for the short term because it takes only 72 h for the Ag^+^ to completely leach out from PMMA film [60]. Even though we have not studied further about the efficacy of our AgNPs in PCL over time, it could be expected that Ag^+^ will be released from PCL/AgNPs scaffold using a similar mode of action with PMMA/AgNPs reported by Lyutakov et al. [60]. Thus, this result signifies that PCL/AgNPs composite scaffolds could be expected as one promising blend of material for realizing long-term effective anti-infection properties.

It is also worth noting that the green synthesis approach offers less cytotoxicity of the obtained AgNPs than the conventional physical or chemical synthesis route due to the absence of toxic chemical reducing agents involved during synthesis process and the presence of surface coating/capping agents [71]. Furthermore, PCL/AgNPs blends were prepared by physical melt blending without the use of toxic organic solvents, reducing toxicity of the fabricated PCL/AgNPs scaffolds. Thus, further studies are required to measure cytotoxic properties of the fabricated PCL/AgNPs scaffolds with a human cell model.

This work demonstrates that AgNPs can be produced using green synthesis methods, eliminating the use of harmful chemicals which could affect their performance in bone tissue scaffold applications and also creates a sustainable method to manufacture these nanoparticles. This work also showcases the potential application of AgNPs as a filler material for electroactive polymeric bone scaffold with anti-infection properties due to its inherent electroconductive and antibacterial properties.

## 3. Materials and Methods

### 3.1. Materials

AgNO_3_ and NaOH pro analysis (p.a.) grade were supplied by Merck KGaA, Darmstadt, Germany and used without further purification. Cilembu sweet potatoes were purchased at a local store in Bandung, Indonesia. Demineralized water was obtained from Brataco Bandung, Indonesia. PCL (CAPA 6500, Mw 50,000) supplied by Perstorp, Warrington, UK, is a semi-crystalline linear aliphatic biopolymer with a melting point of 58–60 °C, a glass transition temperature of ~−60 °C, and a density of 1.146 g/mL at 25 °C used to prepare composite blends with AgNPs. *Staphylococcus aureus* (*S. aureus*; ATCC 3658) bacterial strain was obtained from Microbiology Laboratory, School of Pharmacy, Institut Teknologi Bandung (ITB).

### 3.2. Preparation and Characterization of Silver Nanoparticles

#### 3.2.1. Preparation of Extract of Cilembu Sweet Potatoes

Extract of Cilembu sweet potatoes were prepared as both reduction and capping agents following a similar procedure previously proposed by Roy et al. [50]. Two types of Cilembu sweet potatoes samples were used in this study: (i) the unbaked sample is the fresh Cilembu sweet potatoes that were used directly without any treatment and (ii) the baked sample is the fresh Cilembu sweet potatoes that were baked in oven at 120 ± 10 °C for 60 min. Both unbaked and baked Cilembu sweet potatoes were peeled from the skin and cut into small pieces. A 100 g of tuber was added into 500 mL of distilled water and stirred using a hot-plate stirrer at 350 rpm and 100 °C for 10 min. The mixture was settled down and then filtered using filter paper (Whatman No.42, pore size 2.5 µm) to obtain aqueous sweet potato extract. NaOH was added dropwise to adjust the solution’s pH to 8 to achieve the favorable pH for AgNPs formation [72].

#### 3.2.2. Synthesis of Silver Nanoparticles

AgNO_3_ solution was prepared in three different concentrations: 2 mM; 20 mM and 200 mM. The solution was then added to the previously made extract in a 1:1 volume ratio, making the AgNO_3_ final concentrations 1 mM; 10 mM and 100 mM for baked extract samples and 10 mM for the unbaked extract sample. The mixture was then stirred for 10 min in a dark room without natural sunlight, resulting in colloidal samples. These samples were named based on the type of extract and followed by AgNO_3_ final concentrations (designated as Baked 1; Baked 10, Baked 100 and Unbaked 10 respectively).

AgNPs powder was prepared by centrifuging the colloidal samples using a centrifugal machine at 4000 rpm, to produce precipitates. The remaining liquid part was thrown away and replaced with demineralized water to purify the precipitate sample from unnecessary compounds. This process was repeated three times. Afterwards, freeze-drying was employed to obtain the samples in their powder form for further characterizations.

#### 3.2.3. Silver Nanoparticles Characterization

##### UV-Vis Spectroscopy

UV-Vis spectroscopy (Hewlett Packard Agilent Technologies 8453 Series, Santa Clara, CA, USA) was carried out in a wavelength range between 190–800 nm to confirm the presence of AgNPs in the colloidal samples by exploiting their surface plasmon resonance (SPR) properties. The measurements were performed at room temperature. Before measurement, the samples were prepared by diluting five drops of the colloidal samples in two ml of demineralized water and placed in the cuvette for further characterization.

##### X-ray Diffraction

X-Ray diffraction (XRD; Bruker D8 Advance, Mannheim, Germany) method was used to analyze the structural phase of the obtained samples using Cu Kα, λ = 1.54 A, a voltage of 40 kV, and generator current of 35 mA. The scanning scope angle (2θ) was 10–80 degrees. The characterization was performed at room temperature.

##### Ion Chromatography

Ion chromatography (IC; Dionex ICS, Sunnyvale, CA, USA) was carried out to determine the chloride ion concentration in the extract of Cilembu sweet potatoes. Next, 10 μL of the extract sample was introduced to the columns, using an aqueous solution containing 2.7 mM sodium carbonate and 0.3 mM sodium bicarbonate for detecting the anion. The chromatographic procedure was operated at room temperature and 50 Hz acquisition rate.

##### Dynamic Light Scattering and Zeta Potential

The average particle size and zeta potential of nanoparticles in the colloidal samples were examined using the dynamic light scattering (DLS) and zeta sizer method (Beckman Coulter Delsa™ Nano C Particle Analyser, Brea, CA, USA). The DLS method measured the hydrodynamic diameter of particles in the colloidal suspension and particle sizes were reported as intensity particle sizes distribution (PSD). The scattering angle was set to 165 degrees and performed at room temperature (25 °C). Prior to characterization, the colloidal samples were diluted by adding demineralized water with a volume ratio of 1:1, and 2 mL of diluted sample was used for measurement.

##### Transmission Electron Microscopy and Selected Area Electron Diffraction

The morphology and average individual particles size of nanoparticles were observed by transmission electron microscopy (TEM) (Hitachi HT77000, Tokyo, Japan) that was operated at 120 kV and at room temperature. ImageJ software (developed by the National Institutes of Health and the Laboratory for Optical and Computational Instrumentation, University of Wisconsin, USA) was used to analyze the size of nanoparticles. TEM samples were prepared by diluting the colloidal samples with water and sonicating for 30 min to ensure a good dispersion of particles. Then, two drops of each samples were dropped onto the surface of TEM carbon grid and dried at room temperature.

A similar preparation method was used to prepare Baked 10 and Unbaked 10 samples for selected area electron diffraction (SAED) observation, which was performed using high-resolution TEM (HR-TEM, Hitachi H9500, Tokyo, Japan) at 300 kV in room temperature in order to determine its crystallographic structure to pinpoint the sample’s crystalline identity. ImageJ software was used to analyze the obtained SAED pattern by measuring the ring radii, which will then be converted to diffraction angle (θ) and compared with the lattice parameter database.

##### Fourier Transmitted Infra-Red Spectroscopy

Fourier Transmitted Infra-Red Spectroscopy (FTIR) (Prestige 21 Shimadzu, Kyoto, Japan) was used to analyze functional groups of the extract of Cilembu sweet potatoes and the AgNPs powders. The sample powder (10–15 mg) and potassium bromide (KBr) powder (150–250 mg) were mixed and pressed at 700 kN for 5 min to produce a pellet shape. The characterization was performed at room temperature and the FTIR was recorded in the range of 4000–500 cm^−1^.

##### The Disc Diffusion Test

The disc diffusion method was performed to qualitatively evaluate the antibacterial activity of the synthesized AgNPs based on Kirby-Bauer test method [73]. *Staphylococcus aureus* (*S. aureus*; ATCC 3658) bacterial strain was used as the bacterial model. Beforehand, the strains were cultured and presented an absorbance value of 0.11 under UV-Vis spectroscopy observation with a wavelength of 625 nm. Then, the bacterial culture was diluted with NaCl solution with a ratio of 1:20 to obtain a bacterial colony of 10^6^ CFU that will be used for antibacterial testing.

Even though the streak inoculation method is the widely accepted method for testing pure antimicrobial molecules such as antibiotics, Othman et al. found that pour plate disc diffusion (PPDD) test shows the most reproducible results for plant crude extract [74]. Thus, this method was used in this study to obtain smooth growth of bacteria in agar. Briefly, 200 μL of 10^6^ CFU was mixed with 20 mL of Mueller Hinton Agar (MHA) on the petri dishes. The mixture solution was solidified after 15 min. Afterward, the paper discs were put on the agar surface and colloidal samples of 10 μL were dropped on the paper disc. The baked extract was used as a control. Pre-incubation was conducted for 15 min in an open space followed by 24 h incubation at 37 °C in the incubator. This procedure was performed in triplicate.

### 3.3. Fabrication and Characterization of 3D Printed of PCL/AgNPs Scaffold

#### 3.3.1. Fabrication of PCL/AgNPs Scaffolds

Homogeneous PCL/AgNPs blends were prepared by physical melt blending. Briefly, the AgNPs powder (0.5 wt.%) from the Baked 10 sample was added to the melted PCL (10 g) at ~80 °C and the mixture was stirred manually for about 30 min to obtain homogeneous blending. After cooling, the PCL/AgNPs blend was cut into small pellet-sized pieces to facilitate its loading into the 3D printer’s material chamber [6,13].

Scaffolds were fabricated using a screw-assisted extrusion system (3D Discovery, regenHU, Villaz-St-Pierre, Switzerland) with a printing nozzle diameter of 0.5 mm. The computer-aided design software was used to produce the scaffold’s model architecture (BioCAD, regenHU, Villaz-St-Pierre, Switzerland). Scaffolds were designed considering a filament distance of 1 mm, a slice thickness of 280 µm, and a 0°/90° lay-down pattern, with overall dimensions of 20 mm × 20 mm × 3 mm. The following processing conditions were considered to produce the scaffolds: deposition velocity of 5 mm/s, melting temperature of 90 °C, extrusion pressure of 6 bar, and screw rotation velocity of 15 rpm.

#### 3.3.2. Scaffold Characterization

##### Morphology

Scanning electron microscopy (SEM, Hitachi S3000N, Hitachi, Tokyo, Japan) was used to observe the scaffold morphology. Prior to observation, the scaffolds were sputtered by gold coating for 40 s. The imaging acquisition process was carried out using a 10 kV accelerating voltage considering both the scaffolds top-down and cross-sectional sides. ImageJ software was used to determine the pore size and fiber diameter from SEM images.

The porosity of the scaffold was measured using the Archimedes Law [75]. The scaffolds (10 × 10 × 3 mm^3^) were weighed in air (*m*_1_) and liquid ethanol (*m*_2_) (*ρ_eth_* = 789 kg/m^3^), and the volume of the immersed object (*V_t_*) was calculated as follow:

(1) $$F_{a}=\rho_{eth}\times g\times V_{t}$$

where *F_a_* is the upward lift (N), and g is the earth’s gravity (9.8 m/s^2^). Knowing the volume of the scaffold (m^3^), *V_u_*, the porosity can be calculated according to the following equation:

(2) $$Porosity\;\left(\%\right)=\frac{V_{u}-V_{t}}{V_{t}}$$

The test was carried out three times.

##### Wettability

The wettability of scaffolds was assessed using the static water contact angle at room temperature. A ~13 µL demineralized water droplet was dropped onto the scaffold. A high-speed camera captured the droplet and the contact angle calculated using a Young–Laplace fit and shape analysis method (KSV CAM 200, KSV Instruments, Helsinki, Finland).

##### Mechanical Properties

Mechanical properties of the PCL and PCL/AgNPs scaffolds were assessed with compressive tests using a Universal Testing Machine (Tensilon RTF-1310, A&D Company, Tokyo, Japan) at room temperature according to the ISO 844-2014 standard [62]. Briefly, scaffold samples (n = 3) with a dimension ratio of 1:1:1 were prepared by cutting from the 3D printed scaffold, which were then deformed by compression plates with a load cell of 10 kN at a rate of 0.5 mm/min. The load-displacement data was used to calculate the compressive Young’s modulus from the gradient of the initial elastic region on a stress-strain curve, while the compressive strength was defined as compressive stress at 10% strain.

##### Electrical Conductivity

An Alessi four-point probe was used to measure the electrical conductivity of the PCL/AgNPs scaffold samples (n = 3) at room temperature [76]. The two outer probes were used to supply high impedance current (*I*) through the scaffold, and the voltage (*V*) on the other two inner probes were measured using an electrometer. The distance between probes was 1 mm and will be denoted as *L*, while *F* is the correction factors for thickness and geometry of the scaffold samples. The scaffold’s electrical conductivity (*σ*) was calculated as follows:

(3) $$\sigma =\frac{IL}{VF}$$

##### Total Plate Count Method

The scaffolds antibacterial properties were determined by the total plate count method following the procedure reported by Gao et al. [71]. Before testing, the scaffolds were sterilized by an ultraviolet lamp with a specific wavelength (260 nm) for about 15 min [77]. Briefly, a sample of bacteria (*S. aureus*; ATCC 3658) from the sterilized scaffold was taken from the inhibition zone to calculate the decrease of the bacteria colony using serial dilution. The dilution was carried out by adding 100 µL of semi-solid agar around the samples into 900 µL of 0.9% NaCl in each dilution. After six times dilution (10^6^), the colony of bacteria (n) were observed in the ideal number (30 ≤ n ≤ 300). Subsequently, each 1 mL of a 10^−6^ dilution was added to sterile petri dishes followed by the addition of 20 mL of Mueller Hinton Agar (50 °C) medium. The mixture was rotated clockwise and counterclockwise five times until a homogenous mixture was produced. The samples were left to solidify for around 20 min, and then incubated at 37 °C for 24 h. The number of bacteria was observed and counted with a colony counter, Suntex. The procedure was repeated in triplicate for each sample.

## 4. Conclusions

This study aims to produce AgNPs through green synthesis approach and assess the feasibility of the obtained AgNPs as an electroconductive and antibacterial filler in polymeric bone scaffolds. Firstly, the study showed that AgNPs were successfully prepared by a facile green synthesis approach using extract of Cilembu sweet potatoes, without requiring any harmful reducing agent and high temperature/pressure. The results showed that the baking process is crucial in assisting the formation of AgNPs, because it enhances the reduction of sugar content in the extract, which is confirmed by the formation of AgNPs in the baked sample and AgCl in the unbaked sample. Moreover, the employment of ten mM AgNO_3_ and baked extract was found to be the optimum condition to obtain the smallest particle size of AgNPs (9.95 ± 3.69 nm) with spherical-shape morphology and stable colloidal dispersion, which is verified by its zeta potential (−41.0 ± 9.0 mV).

Secondly, the synthesized AgNPs were melt blended with PCL and were 3D printed to produce tissue scaffolds. PCL/AgNPs scaffolds exhibited several enhancements compared to PCL scaffolds. Compressive strength was six times greater (3.88 ± 0.42 MPa) and the scaffolds were more hydrophilic (contact angle of 76.8 ± 1.7°), which are beneficial in functioning as a more suitable mechanical support for cancellous bone and promoting new cell proliferation. In fulfilling its role as an electroactive scaffold, incorporation of AgNPs magnificently imbued electroconductive properties (2.3 ± 0.5 × 10^−3^ S/cm) to the originally non-conductive PCL scaffold. The presence of AgNPs in PCL/AgNPs scaffolds were fruitfully demonstrated the heightened anti-bacterial properties when tested against common infection-causing model bacteria, *S. aureus* ATCC 3658 (99.5% reduction of surviving bacteria). It is also likely that due to the slow release of Ag^+^ from AgNPs and high antibacterial activity of small size of AgNPs with spherical-shape morphology, the fabricated PCL/AgNPs scaffolds will have long-term effective anti-infection properties if used as a biomaterial within an implanted medical device. Despite the promising results, further investigation on antibacterial activity against other strains of bacteria, antimicrobial mechanisms, and cytotoxic properties of the fabricated PCL/AgNPs scaffolds with a human cell model are required to obtain comprehensive study of this scaffold. This green synthesis approach, together with the use of 3D printing, opens a new route to manufacture AgNPs-based electroactive scaffold with improved anti-bacterial properties without the use of any toxic reducing agent and organic solvents.

## Figures and Tables

**Table 1:** Summary of dynamic light scattering (DLS) and zeta potential results of AgNPs samples.

Sample Code	DLS Results		Zeta Potential (mV)
	Average Diameter (nm)	Polydispersity Index	
Baked 1	364.6 ± 95.3	0.25 ± 0.04	−15.5 ± 2.0
Baked 10	105.5 ± 12.6	0.29 ± 0.02	−41.0 ± 9.0
Baked 100	456.5 ± 14.7	0.22 ± 0.01	−0.7 ± 0.4
Unbaked 10	5958.8 ± 499.6	0.29 ± 0.02	−0.3 ± 0.1

**Table 2:** Antibacterial results of samples from disc diffusion method.

Sample	Sample Form	ZOI (mm)
Extract-only	Liquid	0
Baked 1	Powder	0
Baked 10		0
Baked 100		0
Baked 1	Colloidal	11.2 ± 0.3
Baked 10		12.9 ± 0.3
Baked 100		15.1 ± 0.6
Unbaked 10		11.7 ± 0.3

**Table 3:** Summary of average fiber diameter and pore size of PCL and PCL/AgNPs scaffolds based on SEM and porosity based on the Archimedes Law (n = 3).

Scaffold Sample	Fiber Diameter (µm)	Pore Size (µm)	Porosity (%)
PCL	480 ± 76	569 ± 59	70 ± 34
PCL/AgNPs	536 ± 24	425 ± 39	60 ± 4

**Table 4:** Compressive Young’s modulus and compressive strength at 10% strain of the PCL and PCL/AgNPs scaffolds.

Scaffold Sample	Compressive Young’s Modulus (MPa)	Compressive Strength (MPa)
PCL	13.65 ± 2.17	0.65 ± 0.09
PCL/AgNPs	52.42 ± 7.64	3.88 ± 0.42

## Data Availability

The data presented in this study are available on request from the corresponding author. The data are not publicly available due to institutional restrictions.

## Sample Availability

Samples of the compounds are available from the authors.
